# Diet of Two Large Sympatric Teleosts, the Ling (*Genypterus blacodes*) and Hake (*Merluccius australis*)

**DOI:** 10.1371/journal.pone.0013647

**Published:** 2010-10-27

**Authors:** Matthew R. Dunn, Amelia M. Connell, Jeff Forman, Darren W. Stevens, Peter L. Horn

**Affiliations:** National Institute of Water and Atmospheric Research Limited, Wellington, New Zealand; University of California Davis, United States of America

## Abstract

Ling and hake are tertiary consumers, and as a result both may have an important structuring role in marine communities. The diets of 2064 ling and 913 hake from Chatham Rise, New Zealand, were determined from examination of stomach contents. Ling was a benthic generalist, and hake a demersal piscivore. The diet of ling was characterised by benthic crustaceans, mainly *Munida gracilis* and *Metanephrops challengeri*, and demersal fishes, mainly Macrourids and scavenged offal from fishing vessels. The diet of hake was characterised by teleost fishes, mainly macrourids and merlucciids. Multivariate analyses using distance-based linear models found the most important predictors of diet variability were depth, fish length, and vessel type (whether the sample was collected from a commercial or research vessel) for ling, and fish length and vessel type for hake. There was no interspecific predation between ling and hake, and resource competition was largely restricted to macrourid prey, although the dominant macrourid species predated by ling and hake were different. Cluster analysis of average diet of intraspecific groups of ling and hake confirmed the persistent diet separation. Although size is a central factor in determining ecological processes, similar sized ling and hake had distinctly different foraging ecology, and therefore could influence the ecosystem in different ways, and be unequally affected by ecosystem fluctuations.

## Introduction

Ling *Genypterus blacodes* (Forster 1801), and hake *Merluccius australis* (Hutton 1872), are the largest teleosts commonly found in deep water (400–1000 m) fish assemblages around New Zealand, where they are only surpassed in size by a few species of sharks and skates. Both ling and hake support important commercial fisheries, and are caught both as an occasional target species, and a valuable bycatch [1].

Ling and hake are tertiary consumers, occupying the upper trophic levels [2], [3], and as a result both may influence marine communities through top-down (consumer control) interactions [4]. Around New Zealand, the abundance of both ling and hake has declined since the 1980s following increased commercial exploitation [5], [6]. Understanding the trophic interactions of ling and hake is central to understanding their biology, population dynamics, and how changes in their relative abundance or population structure may influence the ecosystem. Research on trophic relationships is an important component in the move towards an ecosystem approach to fisheries management [7].

Ling occur in the southeast and southwest Pacific and the southwest Atlantic, between depths of 100–800 m [2], [8], [9]. On Chatham Rise, there was a substantial longline fishery for ling during the mid-1970s and the mid-1990s, but since the 1980s most ling have been taken in trawl fisheries targeting other species, particularly hoki (*Macruronus novaezelandiae*) [5]. On Chatham Rise, ling reach maximum sizes of about 160 cm (23 kg) [10]. Little is known about the distribution of juvenile ling until they are about 40 cm long, when they begin to appear over most of the adult range [11]. The Chatham Rise ling stock is believed to have declined markedly in the mid-1970s and throughout the 1990s, but has been recovering since about 2000, and stock biomass in 2007–08 was estimated to be about 50% of virgin levels [5]. Ling are the fourth most abundant species by weight in research trawl surveys at depths of 200–800 m on Chatham Rise, accounting for an average of 4% of the total fish catch [12].

Hake occur in the southwest and southeast Pacific and southwest Atlantic, with the Patagonian population often reported as a subspecies *Merluccius australis polylepis* [13], [14]. Hake are widely distributed around New Zealand [15], with juveniles mainly found in inshore regions shallower than 250 m, and adults in depths of 250–1000 m but occasionally as deep as 1400 m. Hake are taken almost exclusively by trawl, usually as bycatch in fisheries targeting hoki or southern blue whiting (*Micromesistius australis*), although target fisheries exist [16]. Hake reach maximum sizes of about 130 cm (18 kg) [14]. The Chatham Rise hake stock was fished down through the 1990s, but stock biomass in 2009–10 was estimated to be about 50% of virgin levels, following strong recruitment in 2002 [6]. Hake are generally in the top fifteen most abundant species by weight in research trawl surveys at depths of 200–800 m on Chatham Rise, accounting for an average of 1% of the total fish catch [12].

Chatham Rise is a submarine ridge which runs eastwards for about 1000 km from the northeast coast of the South Island of New Zealand, rising up from depths of about 3000 m to 50 m at the western end, and sea level at the eastern end. The subtropical front forms over Chatham Rise throughout the year, where warmer subtropical surface water from the north meets colder subantarctic surface water from the south [17], [18]. The subtropical front extends along the length of Chatham Rise and over a wide latitudinal range (∼100 km) [19], with the strongest surface temperature gradients on the southern flank west of about 177°W, then tending northwards between 177°W–180°, and then becoming diffuse and tending southwards past the Chatham Islands [18]. The subtropical front is a region of heightened primary productivity [20], supporting abundant mesopelagic biomass [21], and also acts as an area of pelagic [22], [23], demersal [24], and benthic [25]–[27] ecosystem discontinuity. The demersal fish assemblage on Chatham Rise has the highest species richness found in New Zealand waters, with species richness higher on the northern slope and increasing with depth to a peak at about 1000 m [28]. Because of the influence of the subtropical front, Chatham Rise is expected to provide a variety of environmental conditions and foraging opportunities for ling and hake, which could lead to significant variability in feeding ecology [29], [30].

The primary objective of this research was to determine the degree of predation or resource competition between ling and hake, and thereby consider to what degree the population dynamics of the two species are likely to be independent. A second objective was to examine the influence of species biology and environmental variability on diet.

## Materials and Methods

### Ethics

This study was exempt from ethical approval by the NIWA Animal Ethics Committee.

### Sampling from research surveys

Biological samples of ling and hake were obtained from stratified-random research bottom trawl surveys on Chatham Rise during December 2004-January 2005, December 2005-January 2006, and December 2006-January 2007 [31]. The sampling area consisted of 26 strata defined by location and depth covering 146 855 km^2^ and depths between 200 and 1000 m. The trawl used was a full-wing bottom trawl, which was towed only during daylight hours [31]. Ling and hake were sampled from all tows where they were caught. Any tow catching more than 15 ling was sub-sampled, consisting of a random sample of 10 fish, and then a non-random sample (5 fish) selected to ensure sampling of the full size range. This allowed stomach sampling to be efficiently integrated with existing random biological sampling, but also provided some non-random samples to focus on identifying ontogenetic shifts in diet. All hake caught were sampled. Selected fish were measured (total length (TL) to the nearest mm), weighed (to the nearest 5 g), and sexed. Fish with obviously regurgitated or everted stomachs were not sampled. At sea, stomachs were sealed by fixing a cable-tie around the oesophagus, then the oesophagus was cut in front of the tie, the intestines cut below the pyloric sphincter, and the stomach removed, labelled, frozen at −20°C and returned to the laboratory.

### Sampling from commercial fishing vessels

In order to increase temporal coverage, biological samples of hake and ling were collected by New Zealand Ministry of Fisheries observers aboard commercial fishing vessels, during November 2005, February-July 2006, and April-May and July 2007. All vessels used bottom trawls, at depths of 264–839 m (median 533 m), for 24 hours a day. The location fished was predominantly the north and west Chatham Rise. Fish catches were sampled opportunistically, depending upon other observer duties. If a tow was sampled, then stomach samples were collected (alongside normal biological samples) from up to 20 ling or hake randomly selected from the catch. Selected fish were measured for total length (to the nearest full cm below), weighed (to the nearest 100 g) where facilities allowed, and sexed. Stomachs were removed and returned to the laboratory following the above protocol.

### Laboratory analyses

Each stomach was thawed, the wet weight of the entire stomach and contents recorded to the nearest 0.1 g, the stomach contents removed and rinsed with water using a 500 µm steel sieve, a qualitative assessment made of digestion state (Fresh; Slightly digested (outer/exposed tissues starting to digest); Partially digested (soft tissues breaking down but hard parts complete); Well digested (fragmented hard parts, soft tissues fully digested)), and the wet weight of the empty stomach recorded to the nearest 0.1 g. Recognisable prey items were then identified to the lowest possible taxonomic level, using reference guides and a reference collection of preserved specimens and hard parts held in NIWA, Wellington. For each prey category, the number of prey individuals was estimated, and wet-weight recorded to the nearest 0.01 g after removal of surface water by blotting paper. A fragmented prey count was based on the number of eyes, heads, tails/telsons, or other anatomical parts traceable to a single specimen. Fish prey were recorded as potentially eaten in the trawl net if they appeared very fresh, with no signs of digestion. Fish prey were recorded as potentially scavenged discarded offal if they consisted of only cleanly severed fish heads and/or tails, or filleted fish frames.

### Statistical analyses

To complete analyses of diet variability the prey items were aggregated into taxonomic categories. The taxonomic prey categories were chosen to achieve maximum prey resolution, whilst maintaining sample size. The taxonomic level of each prey category varied with the ability to identify different prey taxa. The unidentifiable prey (including unidentifiable crustaceans, fish or cephalopod remains), sand, rocks, human waste, shell fragments presumably from Mollusca, nematode and trematode parasites found in the stomachs, and prey classified as well digested, were excluded from detailed analyses. A taxonomic categorisation was used because the knowledge of prey ecology was generally poor, and insufficient to allow a convincing ecological grouping of prey.

To assess the adequacy of the samples for the analyses of diet variability, the cumulative number of prey types identified, and cumulative diversity of the categorised stomach contents measured using the Brillouin index of diversity (H), were plotted against the cumulative number of non-empty stomachs [32]. The mean and 95% confidence intervals were calculated from 1000 curves based upon different random orders of the stomachs. The asymptotic diversity of categorised prey (H_A_) was estimated from a fitted curve of the form H = aN/(1+bN), where a and b are constants, N is the number of stomachs sampled, and the asymptote is given by a/b [33]. The sample was considered adequate if the mean sample diversity (H) was more than 95% of the asymptotic diversity (H_A_).

The contribution of different prey items to the diet was determined by the numerical importance (%N), frequency of occurrence (%F), and mass (%W) [34]. The index of relative importance (IRI), was calculated as IRI = %F (%N+%W), and expressed as a percentage (%IRI) [35]. Bootstrap methods, consisting of 1000 replicates of random samples, with replacement, of stomachs from the original data set (i.e., both empty and non-empty stomachs) stratified by vessel type, trip, and tow, were used to estimate 95% confidence intervals around the dietary statistics [36].

Distance-based linear model (DistLM) analysis was used to identify which of the potential predictors explained most of the variability in diet [29], [37]. Data were standardised by expressing the weight of each prey item as a proportion of the total weight in each stomach, then square-root transformed, and a dissimilarity matrix calculated using Bray-Curtis distances [38]. The potential predictors were vessel type (research or commercial), year, month, time of day, fish length (TL), sex, position of the tow in longitude, latitude, and depth (average of tow start and end positions), and two categorical location predictors derived from tow location and prior knowledge of environmental and faunal gradients; west-east and subtropical front (STF). The west-east predictor consisted of west and east strata, split at the 180° longitude [29]. The STF predictor consisted of the categories bank (200–349 m), crest (350–499 m), northern slope (500–800 m), and southern slope (500–800 m) [29]. More detailed information on prey distributions were not available, and more detailed environmental data were not collected by commercial vessels. The results of the DistLM analysis were a marginal test, fitting each predictor individually, and a conditional test, fitting each predictor conditional on the predictor(s) already in the model [37]. The most significant predictors in the conditional tests were selected using the “best” selection method, using the Akaike Information Criterion and Bayesian Information Criterion [37]. Significant and relevant correlations between predictors varied between species samples, and are reported in the results.

To further investigate the effects of the predictors identified from the DistLM analysis, the continuous predictors were binned. Bin limits were chosen so that the number of observations in each bin was approximately equal [33]. This was considered objective given that there were no *a priori* known biologically meaningful boundaries for these predictors, and it prevented bins containing small, and so potentially biased, samples. The target number of samples in each bin was sufficiently large to describe >85% of the estimated diversity of the overall diet. The binned data were averaged (mean of normalised proportions of prey species), square-root transformed, and then analysed using non-parametric multidimensional scaling (MDS), followed by SIMPER (similarity percentages), using PRIMER v6 [39]. Similarity levels were indicated on MDS plots following a cluster analysis using the average linkage method [40], [41]. The SIMPER was used to identify, based on the contribution to the overall Bray-Curtis dissimilarity, which prey species were characteristic of the diet within each bin. The mean percentage contribution of the prey groups identified by SIMPER were plotted to show the main differences in dietary composition between bins.

Dietary overlap was estimated using hierarchical agglomerative clustering [29], [41], and intraspecific groups, to determine whether intraspecific similarities were greater than interspecific similarities. The intraspecific groups were defined from the previous analyses, therefore within each group the diets were similar. To avoid any intraspecific groups containing small, and so potentially biased samples, the groups were determined using only the most important 1 or 2 predictors of diet variability in each species. The data were standardised prey weight averaged within groups, square root transformed, a dissimilarity matrix calculated using Bray-Curtis distances, and cluster analysis performed using the average linkage method [40].

## Results

### *Genypterus blacodes*

**Figure 1:**
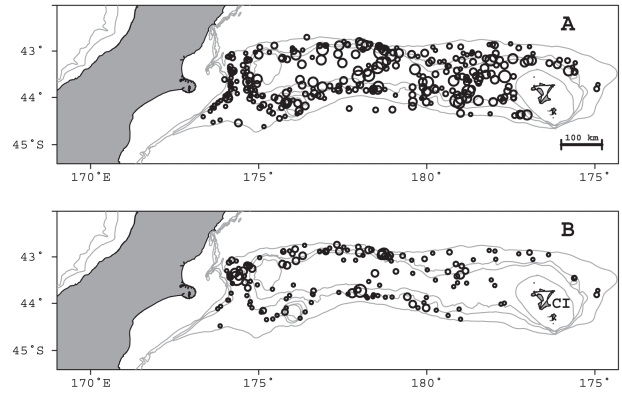
Study area and location of samples. Location of A, ling, and B, hake, stomach samples (circles) on Chatham Rise, New Zealand. Circle area is proportional to sample size (ling max. 15; hake max. 16). Grey lines indicate the 200 m, 350 m, 500 m, and 800 m isobaths. CI, Chatham Islands.

**Figure 2:**
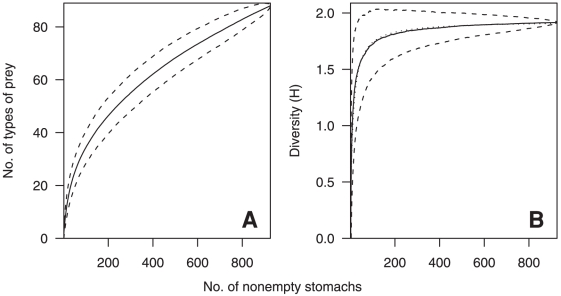
Cumulative prey richness and diversity with increasing ling sample size. Ling number of non-empty stomachs sampled (n = 926) and A, the mean cumulative number of prey types identified, and B, mean cumulative diversity of prey categories (measured using the Brillouin index of diversity, H). Broken lines indicate the 95% confidence intervals. Dotted line in B is a fitted curve from which asymptotic diversity was estimated. Stomachs containing all unidentifiable or well-digested prey were excluded.

Ling were sampled over a wide spatial area and depths of 255–791 m (Fig. 1). Of 2064 specimens examined, 1540 (74%) contained prey. A total of 5273 individual prey of 111 prey groups were identified, having a total weight of 36.8 kg (Appendix S1). The number of prey items per stomach varied between 1 and 37, with 85% of stomachs containing less than 5 prey items, and 50% containing only a single prey item. Prey remains were all unidentifiable or well digested in 614 stomachs, leaving 926 (45%) for detailed analyses of diet (Appendix S1). These specimens had a median length of 80 cm TL (range 33–150 cm TL), and a length (cm) weight (g) relationship of W = 0.0013×TL^3.287^ (*n* = 859; r^2^ = 0.99). The mean length of ling sampled from commercial vessels was significantly larger than that from research vessels (mean lengths 87.6 cm and 76.3 cm TL respectively; t-test, t = 1.97, P≤0.001), although the proportions of large fish (>100 cm TL) were similar at 7% and 6% respectively. New types of prey continued to be identified with increasing sample size, however, the diversity of prey categories reached 95% of the estimated asymptote after 206 stomachs (Fig. 2), indicating that the sample was large enough to describe the diversity of the diet when using the assumed prey categorisation.

The diet of ling was diverse, but characterised by benthic crustaceans and demersal fishes (Appendix S1). Galatheids (mainly *Munida gracilis*) occurred in 50% of stomachs but were relatively small and so contributed only 7% of prey weight. *Metanephrops challengeri* were a relatively large crustacean prey, and contributed a similar weight to galatheids despite occurring in only 9% of stomachs. The fish prey included benthic species, such as eels and flatfish, demersal species such as hoki, mesopelagic species such as myctophids, and 3 instances of cannibalism, but the most important fish prey by %IRI were demersal macrourids, which were found in 17% of stomachs, contributed 16% of prey weight, and consisted of at least 7 species. The greatest %W was from discarded fish remains (30%), which were predominantly severed heads and/or tails of the pelagic jack mackerel *Trachurus* spp., or heads of other fishes with no other accompanying remains, e.g., one stomach contained only 4 hoki heads. The presence of human waste (a lamb chop) reaffirmed opportunistic scavenging behaviour. One stomach contained numerous teleost eggs, but as these occurred along with other fish internal organs it is likely they were from an ingested ovary (possible fish discards) rather than direct predation on teleost eggs. In two stomachs the fish prey were suspected to have been eaten in the net.

The DistLM analysis indicated significant relationships between diet and several of the predictors, with the most parsimonious conditional model having the predictors depth, fish length, and vessel type (Table 1). This model explained 11.7% of the deviance, indicating most of the variability in diet could not be explained by the predictors. There was only a weak correlation between fish length and depth (r^2^ = 0.20).

**Table 1:** Ling results of the DistLM analysis marginal models, and the most parsimonious conditional model chosen using the “best” selection method.

Predictor	d.f.	*P*	*r^2^*
**Marginal model**			
Vessel type	2	0.001	0.034
Year	4	0.001	0.016
Month	5	0.001	0.025
Longitude	2	0.001	0.011
Latitude	2	0.002	0.004
Depth	2	0.001	0.069
Duration	2	0.001	0.030
Time of day	2	0.218	0.001
Fish length	2	0.001	0.056
Sex	2	0.006	0.005
STF	4	0.001	0.064
West-east	2	0.001	0.009
**Conditional (sequential) model**			
Depth	2	0.001	0.069
+ Fish length	3	0.001	0.109
+ Vessel type	4	0.001	0.117

**Figure 3:**
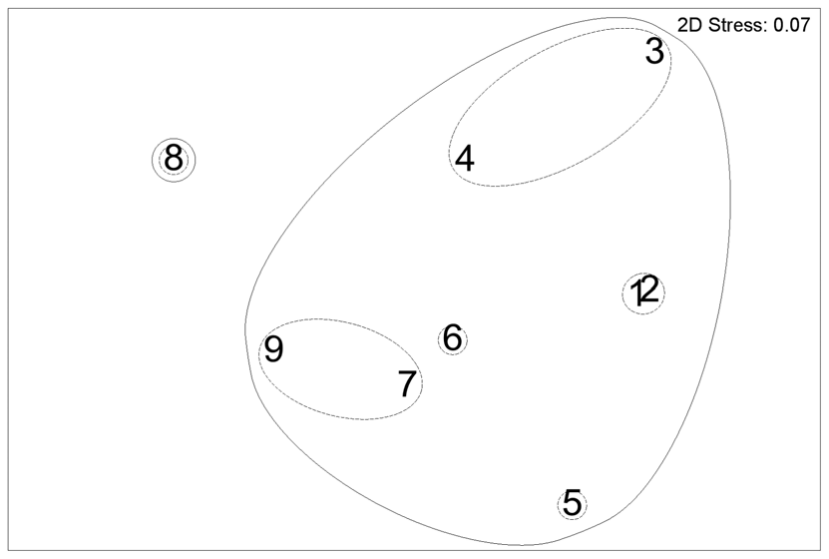
Ling diet, non-parametric multi-dimensional scaling ordination for depth groups. Based on percentage by weight (%W) of diet, for depth groups: 1, 255–353 m (n = 99); 2, 354–381 m (n = 102); 3, 382–410 m (n = 102); 4, 411–428 m (n = 102); 5, 429–452 m (n = 100); 6, 453–483 m (n = 103); 7, 484–514 m (n = 109); 8, 515–559 m (n = 104); 9, 562–791 m (n = 105). Outer line indicates 40% similarity, inner line 60% similarity.

**Figure 4:**
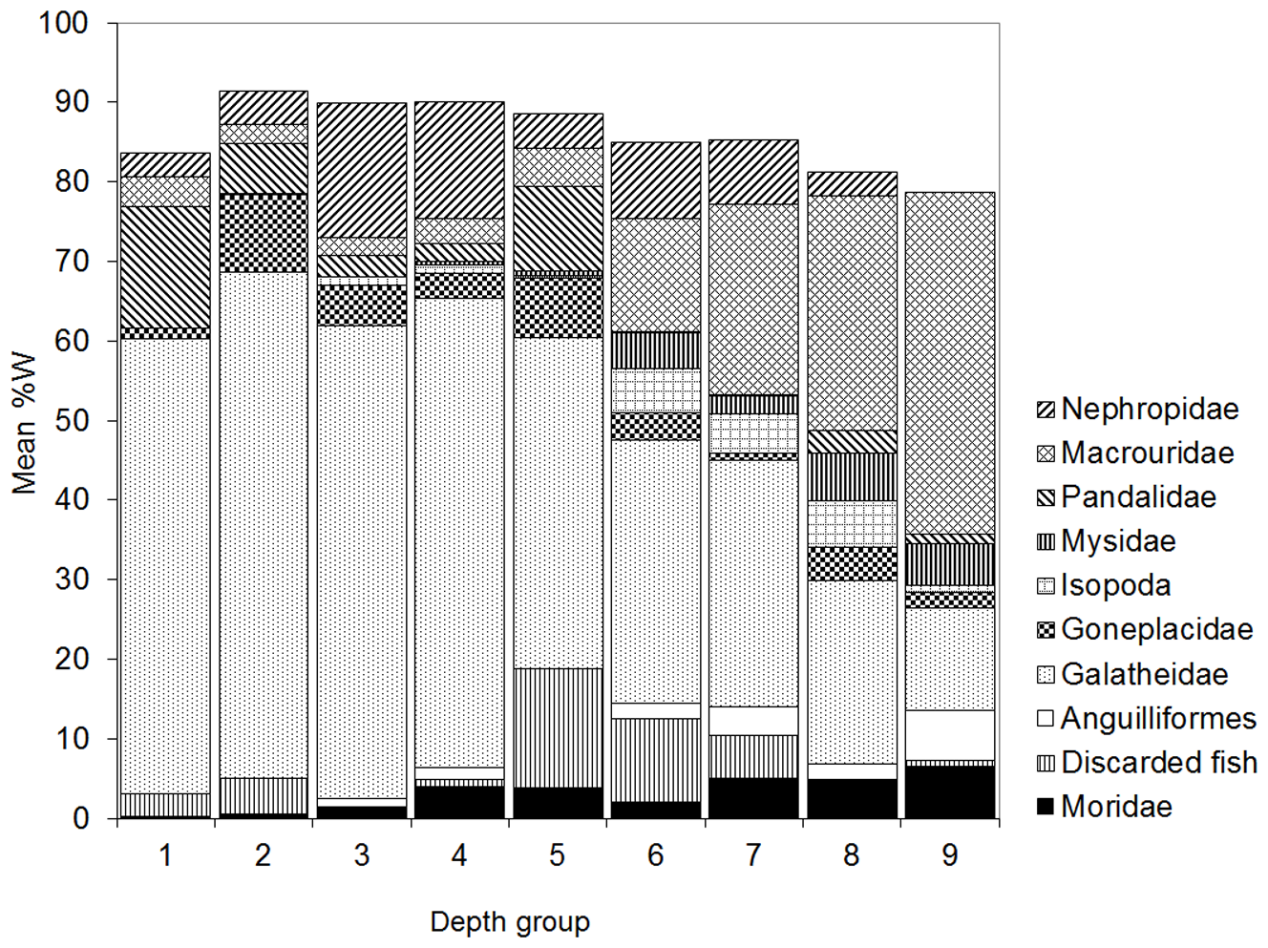
Ling diet by depth group. Contribution of the characteristic prey types to the diet (mean of individual stomach %W) in the depth groups: 1, 255–353 m; 2, 354–381 m; 3, 382–410 m; 4, 411–428 m; 5, 429–452 m; 6, 453–483 m; 7, 484–514 m; 8, 515–559 m; 9, 562–791 m. Prey types shown are those indicated by SIMPER to be characteristic of the diet, having explained at least 90% of the SIMPER within each group.

The MDS plot for depth indicated similar diets at depths 255–381 m, 382–428 m, and 429–791 m but with 515–559 m an outlier (Fig. 3). By prey weight, Galatheidae, Pandalidae, and Goneplacidae were most important in the diet at depths of 255–381 m; Galatheidae and Nephropidae were most important at depths of 382–428 m; Galatheidae, Nephropidae, Macrouridae, Mysidae, and discarded fishes were most important at depths of 429–791 m, with fish prey dominant (mean %W>50%) at 562–791 m (Fig. 4).

**Figure 5:**
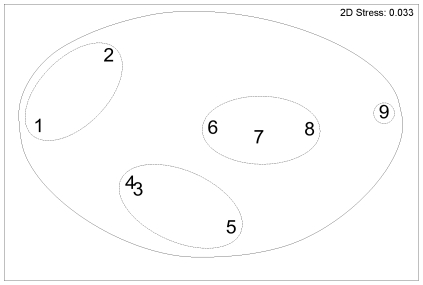
Ling diet, non-parametric multi-dimensional scaling ordination for fish length groups. Based on percentage by weight (%W) of diet, for fish length (TL) groups: 1, 32.7–48.5 cm (n = 103); 2, 48.6–58.8 cm (n = 104); 3, 58.9–66.9 cm (n = 103); 4, 67.0–75.6 cm (n = 103); 5, 75.8–82.9 cm (n = 101); 6, 83.0–89.0 cm (n = 101); 7, 89.3–94.9 cm (n = 102); 8, 95.0–103.6 cm (n = 104); 9, 103.8–149.7 cm (n = 105). Outer line indicates 40% similarity, inner line 60% similarity.

**Figure 6:**
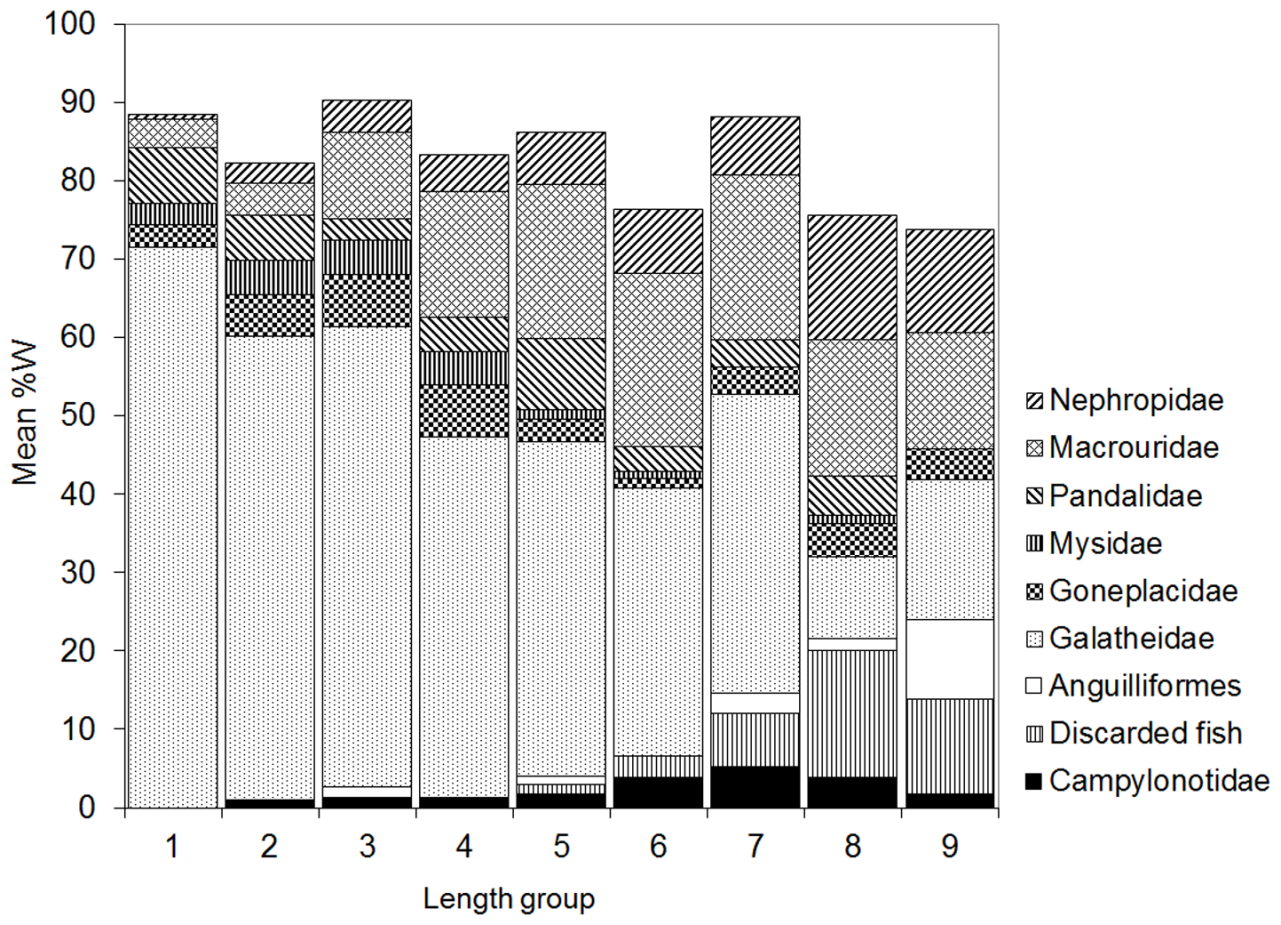
Ling diet by fish length group. Contribution of the characteristic prey types to the diet (mean of individual stomach %W) in the fish length (TL) groups: 1, 32.7–48.5 cm; 2, 48.6–58.8 cm; 3, 58.9–66.9 cm; 4, 67.0–75.6 cm; 5, 75.8–82.9 cm; 6, 83.0–89.0 cm; 7, 89.3–94.9 cm; 8, 95.0–103.6 cm; 9, 103.8–149.7 cm. Prey types shown are those indicated by SIMPER to be characteristic of the diet, having explained at least 90% of the SIMPER within each group.

The MDS plot for fish length indicated similar diets at 32.7–58.8 cm, 58.9–82.9 cm, 83.0–103.6 cm, and 103.8–149.7 cm (Fig. 5). By prey weight, Galatheidae and Pandalidae were most important in the diet of smaller ling of 32.7–58.8 cm; Galatheidae, Pandalidae, Goneplacidae, and Macrouridae were most important in intermediate sized ling of 58.9–82.9 cm; Galatheidae, Nephropidae, Macrouridae, and discarded fish were most important in the larger ling of 83.0–149.7 cm, with Anguilliformes also important at 103.8–149.7 cm (Fig. 6).

Crustacean prey were more important in the diet of ling sampled from research vessels; in research vessel samples Galatheidae and Nephropidae had a mean %W of 47% and 7% respectively (combined contribution to SIMPER within-group similarity of 91%), compared to 18% and 6% in samples from commercial vessels (combined contribution to SIMPER within-group similarity of 23%). Fish prey, in particular Macrouridae, were more important in diet sampled from commercial vessels (mean %W = 33%; contributing 68% to the SIMPER within-group similarity) compared with research vessel samples (mean %W = 10%; contributing 4% to the SIMPER within-group similarity). Vessel type was strongly correlated with tow duration (r^2^ = 0.94), and weakly correlated with longitude (r^2^ = 0.39) and STF (r^2^ = 0.36).

### *Merluccius australis*

**Figure 7:**
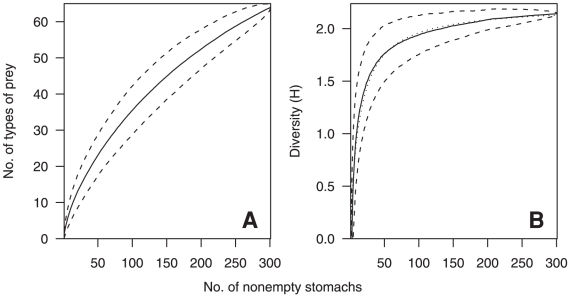
Cumulative prey richness and diversity with increasing hake sample size. Hake number of non-empty stomachs sampled (n = 301) and the A, mean cumulative number of prey types identified, and B, mean cumulative diversity of prey categories (measured using the Brillouin index of diversity, H). Broken lines indicate the 95% confidence intervals. Dotted line in B is a fitted curve from which asymptotic diversity was estimated. Stomachs containing all unidentifiable or well-digested prey were excluded.

Hake were sampled over a wide spatial area and depths of 344–864 m (Fig. 1). Of 913 specimens examined, 677 (74%) contained prey. A total of 1211 individual prey in 71 prey groups were identified, having a total weight of 42.7 kg (Appendix S2). The number of prey items per stomach varied between 1 and 6, with 72% of stomachs containing only a single prey item and only 4% containing more than 3 items. Prey remains were all unidentifiable or well digested in 376 stomachs, leaving 301 (33%) for detailed analyses of diet (Appendix S2). These specimens had a median length of 72 cm TL (range 39–131 cm TL), and a length (cm) weight (g) relationship of W = 0.0021×TL^3.283^ (*n* = 275 r^2^ = 0.99). The mean length of hake sampled from commercial vessels was significantly larger than that from research vessels (mean lengths 79.7 cm and 72.0 cm TL respectively; t-test, t = 1.97, p≤0.001), although the proportions of large fish (>100 cm TL) were similar at 12% and 10% respectively. New types of prey continued to be identified with increasing sample size, however, the diversity of prey categories reached 95% of the estimated asymptote after 231 stomachs (Fig. 7), indicating that the sample was large enough to describe the diversity of the diet when using the assumed prey categorisation.

The diet of hake was dominated by teleost fishes, in particular Macrouridae (Appendix S2). Macrouridae accounted for 44% of the prey weight and consisted of at least six species, of which javelinfish, *Lepidorhynchus denticulatus*, was most frequently identified. Merlucciidae, which were entirely hoki, were less frequent prey, but being relatively large accounted for 37% of prey weight. Many of the fish prey were classified as potentially eaten in the net, but for most species some digested individuals were also found; the only species where all individuals were classified as eaten in the net were *Coryphaenoides serrulatus*, *Halargyreus johnsonii*, and *Epigonus lenimen*. Various squids (Teuthoidea) were found in 7% of the stomachs, and accounted for 5% of the prey weight. Crustacean prey were predominantly natant decapods, and of these pasiphaeid prawns were most frequently found, occurring in 19% of the stomachs, although natant decapods accounted for <1% of the prey weight.

The DistLM analysis indicated significant relationships between diet and several of the predictors, with the most parsimonious conditional model having the predictors vessel type and fish length, and explaining 8.3% of the deviance (Table 2).

**Table 2:** Hake results of the DistLM analysis marginal models, and the most parsimonious conditional model chosen using the “best” selection method.

Predictor	d.f.	*P*	*r^2^*
**Marginal model**			
Vessel type	2	0.001	0.055
Year	4	0.001	0.046
Month	3	0.004	0.021
Longitude	2	0.010	0.012
Latitude	2	0.001	0.019
Depth	2	0.010	0.011
Duration	2	0.001	0.047
Time of day	2	0.004	0.015
Fish length	2	0.001	0.039
Sex	2	0.001	0.023
STF	3	0.001	0.031
West-east	2	0.113	0.006
**Conditional (sequential) model**			
Vessel type	2	0.001	0.055
+ Fish length	3	0.001	0.083

In the samples from commercial vessels, Macrouridae, Merlucciidae and Pasiphaeidae contributed >90% to the within-group SIMPER, and the average percentage prey by weight was 66% Macrouridae, 17% Merlucciidae and 4% Pasiphaeidae, compared to 32%, 8% and 21% respectively in the research vessels. Vessel type was strongly correlated with tow duration (r^2^ = 0.94), and weakly correlated with latitude (r^2^ = 0.31).

**Figure 8:**
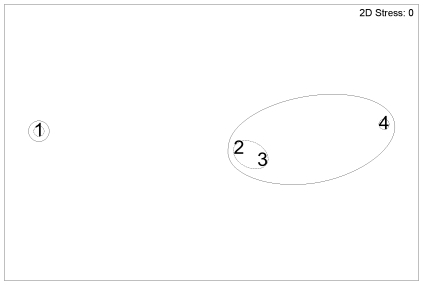
Hake diet, non-parametric multi-dimensional scaling ordination for fish length groups. Based on percentage by weight (%W) of diet, for fish length (TL) groups: 1, 38.5–60.3 cm (n = 76); 2, 60.7–71.5 cm (n = 76); 3, 71.6–85.5 cm (n = 75); 4, 85.7–131.0 cm (n = 74). Outer line indicates 60% similarity, inner line 80% similarity.

**Figure 9:**
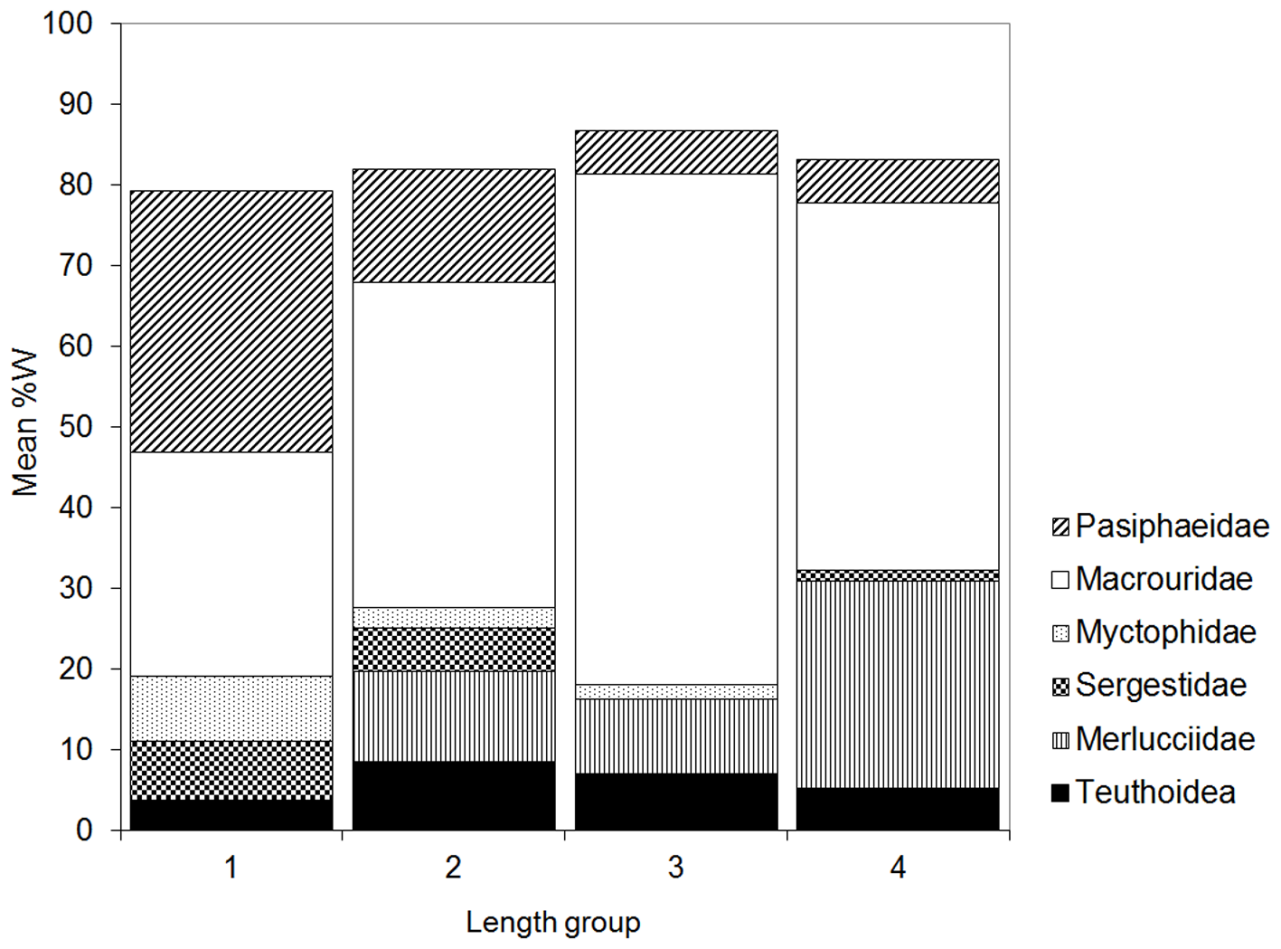
Hake diet by fish length group. Contribution of the characteristic prey types to the diet (mean of individual stomach %W) in the fish length (TL) groups: 1, 38.5–60.3 cm; 2, 60.7–71.5 cm; 3, 71.6–85.5 cm; 4, 85.7–131.0 cm. Prey types shown are those indicated by SIMPER to be characteristic of the diet, having explained at least 90% of the SIMPER within each group.

The MDS plot for fish length indicated a similar diet at 60.7–85.5 cm, which had greater similarity to 85.7–131.0 cm, than to 38.5–60.3 cm (Fig. 8). By prey weight, Pasiphaeidae, Sergestidae, Macrouridae, and Myctophidae were most important in the diet of smaller hake of 38.5–60.3 cm; Macrouridae, Merlucciidae and Teuthoidea were more important, and Pasiphaeidae and Myctophidae less important, in the diet of intermediate sized hake of 60.7–85.5 cm; Merlucciidae were most important in the diet of large hake of 85.7–131.0 cm, with Macrouridae also important (Fig. 9).

### Dietary overlap

**Figure 10:**
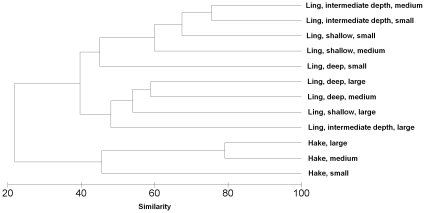
Dendrogram showing the similarity in diet between ling and hake subgroups. Dendrogram of group-averaged cluster analysis of Bray-Curtis dissimilarities based on square root transformed diet %W by subgroup. Shallow, 255–381 m; intermediate depth, 382–428 m; deep, 429–791 m. Ling: small, 32.7–58.8 cm; medium, 58.9–82.9 cm; large, 83.0–149.7 cm. Hake: small, 38.5–60.3 cm; medium, 6.7–85.5 cm; large, 85.7–131.0 cm.

Ling were split into 9 groups; the permutations of the depth groups shallow (255–381 m), intermediate (382–428 m) and deep (429–791 m), and fish length groups small (32.7–58.8 cm), medium (58.9–82.9 cm) and large (83.0–149.7 cm). Hake were split into 3 groups, the fish length groups small (38.5–60.3 cm), medium (60.7–85.5 cm) and large (85.7–131.0 cm). The first split in the cluster analysis was by species, at 22% similarity (Fig. 10). The greatest similarity was between medium and large sized hake (79%). Large ling, and medium sized ling in deep water, clustered together at 48% similarity. The remaining groups of small and medium ling clustered together at 45% similarity.

## Discussion

Size is undoubtedly a central factor in determining ecological processes, but similar sized ling and hake on Chatham Rise were found to have distinctly different foraging ecology. The cluster analysis showed that intraspecific diet similarities were greater than interspecific diet similarities. Neither species appeared to predate on the other. Resource competition was largely restricted to macrourid prey, but the dominant macrourid prey species were different in ling and hake. Ling was a benthic generalist, with a wide range of potential prey, including scavenging; the broad diet makes ling a potential keystone species. Hake was a demersal piscivore, with an apparent preference for smaller silver fish prey such as hoki, *L. denticulatus*, and *Micromesistius australis* [3], [42]. There appears to be intraguild predation between hake and hoki, the dominant fish species on Chatham Rise, because hake competes with hoki for mesopelagic crustaceans and fishes as a juvenile, and then predates hoki as an adult [43], [44]. Because of the different diets, changes in the abundance of ling and hake, brought about by exploitation for example, could have quite different effects on the ecosystem. Ling and hake may also be unequally affected by ecosystem fluctuations or modification, in particular, the benthic foraging of ling makes it potentially vulnerable to any negative impacts of bottom trawling on benthic fauna. Ling are benthic generalists however, and their ability to switch prey, for example to include scavenged material, may help to mitigate this potential effect. The biomass of both ling and hake stocks on Chatham Rise were depleted to about half of the pre-fishing biomass levels by the mid-2000s, following reasonably similar exploitation histories [5], [6]. In recent years ling and hake stocks on Chatham Rise have shown signs of recovery [5], [6], suggesting that the food resources necessary to support them have not, as yet, been substantially degraded as a consequence of fishing disturbance.

We used the DistLM analysis method to efficiently determine the potential importance of a relatively wide range of predictors of diet variability, and avoid making *a priori* judgements about which predictors would have the greatest influence on diet. The choice of prey categorisation used in the analyses was a pragmatic one, and a compromise between achieving sufficient prey resolution to identify diet changes, and maintaining sample sizes. As taxonomic prey resolution increases (i.e., towards genus or species level), any prey that cannot be identified to that level are excluded from analyses and, where these are the only prey in the stomach, this removes the entire stomach from the sample. Had we used a coarse prey classification (e.g., phylum or order level), then more of the stomach samples would have been used, but some diet shifts would not have been identified, for example the transition from Galatheidae to Nephropidae with increasing ling size. Conversely, the failure to classify and analyse other prey at a more detailed taxonomic level might have concealed some changes in diet. Prey were not always identified to a high level simply because digestion had eroded the key characteristics necessary for visual identification. In studies where sample sizes will be small and visual identification a problem, alternative prey identification methods, such as DNA barcoding, might be beneficial [45]. Although the sample sizes in this study were not large enough for us to have encountered sufficient identifiable prey to confidently complete analyses of prey at a genus or species level, and thereby identify the full extent of diet overlap and shifts, we believe our study nevertheless identified the most important aspects of diet content and variability.

Although many environmental predictors were tested, only depth was selected in the final sequential DistLM for ling, with no environmental predictors selected for hake. For ling, both fish size and depth were important predictors of dietary variation: the DistLM indicated depth was the best predictor, although the cluster analysis suggested that ling size was more important. Published information on the depth distribution of many ling prey species are poor or absent. Galatheidae were most important in the diet of ling at depths of 255–428 m, and *M. gracilis* has been found predominantly on the crest and shallower flanks of Chatham Rise at depths of 237–602 m [25]. The scampi, *M. challengeri*, was most important in the diet of ling at 382–514 m, which is similar to the depths at which the commercial fishery targets scampi (300–500 m [1]). Macrourids increased in importance in the diet of ling with increasing depth from 453 m, and the most frequent prey species, *Coelorinchus oliverianus* and *L. denticulatus,* increase in abundance below about 400 m [24]. The changes in ling diet with depth seem reasonable, and appear likely to be a direct response to changes in prey availability.

Changes in diet with ontogeny are ubiquitous in fishes [46]. In ling, the overall diet and ontogenetic shift in diet was similar to that reported in previous studies around New Zealand [8], [47], Tasmania [48], and the Falkland Islands [9]. In hake, the predominance of merlucciid and macrourid prey and the ontogenetic shift in diet was similar to that reported for *M. a. polylepis* around South America [3], and to qualitative descriptions of hake diet in New Zealand waters [42]. Cannibalism has often been reported in other hake species (e.g., [49]), including *M. a. polylepis* off South America [50], but was not found in this study. The key feature of the ontogenetic diet shift in ling and hake was an increase in piscivory with increasing fish size. The size of fish eaten by hake also increased with increasing hake size, as it included myctophids in small hake, macrourids in intermediate sized hake, and hoki in large hake. Similarly, the relatively large Anguilliform fishes were only important in the diet of large ling. The ontogenetic diet shifts in ling and hake were therefore consistent with gape-size limited predation [51]. Scavenging was most pronounced in intermediate sized ling. Scavenging may be less frequent in smaller individuals because they are less able to forage widely, and cannot as easily ingest animal remains such as discarded offal [52]–[55]. Scavenging by ling has only previously been suspected around the Falkland Islands [9], which suggests ling are facultative scavengers. The presence of only severed heads or tails of pelagic mackerel (*Trachurus* spp.), a prey species that would not normally be available to demersal ling, seems convincing evidence of scavenging. For some ling prey, such as hoki, both scavenging and direct predation were recorded. Discarded fish were less important in the largest ling, and Macrouridae and Anguilliformes more important; however both Macrouridae and Anguilliformes would be an unwanted by-catch of commercial fishing, so it is possible they could have been live prey and/or scavenged discards. Scavenging of discarded offal could provide a substantial positive feedback from the commercial fishery to the ling population [56]. Predation of wounded crustacean and fish escapees from trawl nets could provide a further positive feedback [57], for ling and also potentially for hake. The rarity of discarded prey in hake suggested hake had a preference for live prey, or that discarded offal sinks to the bottom where hake to not forage, or where benthic scavengers are more effective. This distinction is reflected in the fisheries on Chatham Rise, where ling are caught on baited long lines but hake seldom are [5], [6], [16]. However, *M. a. polylepis* has been caught on artisanal longlines around South America [58], suggesting this preference is not exclusive.

Stomachs sampled from commercial vessel catches contained more fish prey, which were predominantly macrourids in ling, and macrourids and hoki in hake. The predictor vessel type was correlated with tow length (commercial tows were longer), but this should not have increased the importance of fish prey unless both ling and hake were capable of feeding on fish prey in the net for extended periods (i.e., longer than 50 minutes; the standard duration of a research tow). This seems unlikely and, in addition, fish identified as potentially eaten in the net were relatively infrequent in hake, and rare in ling. Although the ling and hake sampled from commercial vessels were larger, the difference in mean length between vessel types was relatively small (differed by 11.3 cm and 7.7 cm respectively), and the proportion of large fish (>100 cm TL) was similar (differed by 1% and 2% respectively), compared to the difference in %W of fish prey (commercial vessels more than double research vessels). This suggests the difference in length was not sufficient to explain the difference in piscivory. Alternatively, vessel type may have been aliasing for location. Most of the commercial vessels were targeting hoki. Hoki feed predominantly on mesopelagic decapods and fishes, including macrourids [43]. Most macrourids are demersal or benthic feeders, but the most important macrourid prey for ling and hake, *C. oliverianus* and *L. denticulatus*, feed predominantly on mesopelagic prey [30]. A greater importance of fish prey, specifically hoki, *C. oliverianus* and *L. denticulatus*, suggests that commercial vessels may have been focusing their fishing effort in areas where mesopelagic biomass was concentrated, presumably because these were areas where the main target species, hoki, was also concentrated. The vessel type predictor was correlated with spatial predictors (latitude, longitude, or STF), supporting the hypothesis that vessel type might be aliasing for a spatial effect. However, the model preference for the predictor vessel type suggested that the spatial patterns were more complex than individual latitudinal, longitudinal, or STF gradients. Although the reason for the difference between samples from research and commercial vessels remains somewhat obscure, it is important to recognise that substantial differences in diet descriptions may arise from samples collected from different fishing methods.

## Supporting Information

Appendix S1 Ling stomach contents from the Chatham Rise.
(0.27 MB DOC)

Appendix S2 Hake stomach contents from the Chatham Rise.
(0.19 MB DOC)
