# Supplementary material for: Diet of Two Large Sympatric Teleosts, the Ling (Genypterus blacodes) and Hake (Merluccius australis)
Source: PLoS One. 2010 Oct 27;5(10):e13647. doi: 10.1371/journal.pone.0013647 (PMC2965093; doi:10.1371/journal.pone.0013647)
Supplement: Appendix S1 — Ling stomach contents from the Chatham Rise. (0.27 MB DOC) [file pone.0013647.s001.doc]

**Appendix S1.** Ling stomach contents from the Chatham Rise.

|  | %F | %W | %N | %IRI |
| --- | --- | --- | --- | --- |
| **Foraminifera** | **0.32 (0.00–0.83)** | **<0.01 (0.00–0.01)** | **0.12 (0.00–0.30)** | **<0.01 (0.00–0.01)** |
| Foraminifera unidentified | 0.2 | <0.01 | 0.06 | <0.01 |
| **Salpida** | **0.54 (0.10–1.20)** | **0.04 (<0.01–0.12)** | **0.32 (0.03–0.82)** | **<0.01 (<0.01–0.02)** |
| *Iasis zonaria* | 0.07 | <0.01 | 0.02 | <0.01 |
| Salpida unidentified | 0.26 | 0.03 | 0.13 | <0.01 |
| **Mollusca** |  |  |  |  |
| **Gastropoda** | **0.54 (0.00–1.24)** | **0.01 (0.00–0.02)** | **0.24 (0.00–0.55)** | **<0.01 (0.00–0.02)** |
| Gastropoda unidentified | 0.39 | 0.01 | 0.13 | <0.01 |
| **Bivalvia** | **0.11 (0.00–0.45)** | **<0.01 (0.00–0.01)** | **0.04 (0.00–0.17)** | **<0.01 (0.00–0.01)** |
| Bivalvia unidentfiied | 0.13 | <0.01 | 0.04 | <0.01 |
| **Cephalopoda** | **1.62 (0.67–2.78)** | **3.77 (0.20–11.30)** | **0.63 (0.26–1.13)** | **0.18 (0.01–0.68)** |
| Sepiida |  |  |  |  |
| *Sepioloidea* spp. | 0.07 | 0.08 | 0.02 | <0.01 |
| Sepiolidae | 0.07 | <0.01 | 0.02 | <0.01 |
| Teuthoidea |  |  |  |  |
| *Nototodarus* spp. | 0.13 | <0.01 | 0.04 | <0.01 |
| Teuthoidea unidentified | 0.2 | 0.01 | 0.06 | <0.01 |
| Cephalopoda unidentified | 0.39 | 0.01 | 0.11 | <0.01 |
| Octopoda |  |  |  |  |
| *Graneledone taniwha* | 0.07 | 2.67 | 0.04 | 0.01 |
| Octopoda unidentified | 1.11 | 0.67 | 0.32 | 0.06 |
| **Polychaeta** | **1.73 (0.78–2.93)** | **0.01 (0.01–0.02)** | **0.67 (0.29–1.17)** | **0.03 (0.01–0.09)** |
| Eunicidae | 0.07 | <0.01 | 0.02 | <0.01 |
| Polynoidae | 0.13 | <0.01 | 0.04 | <0.01 |
| Terebellidae | 0.07 | <0.01 | 0.02 | <0.01 |
| Polychaeta unidentified | 0.85 | 0.01 | 0.27 | 0.01 |
| **Crustacea** |  |  |  |  |
| **Copepoda** | **0.22 (0.00–0.69)** | **<0.01 (0.00–0.01)** | **0.12 (0.00–0.40)** | **<0.01 (0.00–0.01)** |
| Copepoda unidentified | 0.13 | <0.01 | 0.06 | <0.01 |
| **Euphausiacea** | **0.11 (0.00–0.45)** | **<0.01 (0.00–0.01)** | **0.04 (0.00–0.17)** | **<0.01 (0.00–0.01)** |
| Euphausiacea unidentified | 0.07 | <0.01 | 0.02 | <0.01 |
| **Natant Decapoda** |  |  |  |  |
| **Campylonotidae** | **4.54 (2.97–6.35)** | **0.27 (0.13–0.51)** | **1.81 (1.11–2.65)** | **0.24 (0. 09–0.48)** |
| *Campylonotus rathbunae* | 2.74 | 0.25 | 0.87 | 0.16 |
| **Crangonidae** | **4.86 (3.17–6.67)** | **0.03 (0.02–0.06)** | **2.17 (1.38–3.04)** | **0.27 (0.12–0.51)** |
| *Metacrangon knoxi* | 0.07 | <0.01 | 0.02 | <0.01 |
| *Prionocrangon curvicaulis* | 0.07 | <0.01 | 0.02 | <0.01 |
| Crangonidae unidentified | 2.8 | 0.03 | 1.01 | 0.15 |
| **Nematocarcinidae** | **0.65 (0.10–1.51)** | **0.15 (0.01–0.42)** | **0.28 (0.03–0.65)** | **0.01 (<0.01–0.04)** |
| *Lipkius holthuisi* | 0.39 | 0.13 | 0.13 | 0.01 |
| **Pandalidae** | **8.32 (5.18–11.55)** | **0.24 (0.11–0.47)** | **6.63 (3.90–9.62)** | **1.43 (0.51–2.96)** |
| *Notopandalus magnoculus* | 5.02 | 0.22 | 3.19 | 0.89 |
| **Pasiphaeidae** | **–** | **–** | **–** | **–** |
| *Pasiphaea* spp. | 0.07 | <0.01 | 0.02 | <0.01 |
| **Astacidea** |  |  |  |  |
| **Nephropidae** | **8.65 (5.84–11.65)** | **7.43 (3.99–13.20)** | **4.42 (2.94–6.37)** | **2.57 (1.08–5.02)** |
| *Metanephrops challengeri* | 5.94 | 7.05 | 2.35 | 2.9 |
| **Palinura** |  |  |  |  |
| **Polychelidae** | **0.22 (0.00–0.67)** | **0.02 (0.00–0.06)** | **0.08 (0.00–0.25)** | **<0.01 (0.00–0.01)** |
| *Polycheles* spp. | 0.13 | 0.02 | 0.04 | <0.01 |
| **Anomura** |  |  |  |  |
| **Galatheidae** | **50.38 (45.34–55.53)** | **7.24 (4.57–11.80)** | **54.71 (47.10–61.16)** | **78.29 (69.20–84.26)** |
| *Munida gracilis* | 24.07 | 5.07 | 19.63 | 30.88 |
| *Munida* spp. | 10.37 | 1.07 | 6.33 | 3.99 |
| *Munidopsis kaiyoae* | 0.07 | <0.01 | 0.02 | <0.01 |
| Galatheidae unidentified | 2.94 | 0.52 | 1.57 | 0.32 |
| **Paguridae** | **–** | **–** | **–** | **–** |
| Paguridae unidentified | 0.07 | <0.01 | 0.02 | <0.01 |
| **Paraguridae** | **0.11 (0.00–0.49)** | **<0.01 (0.00–0.01)** | **0.04 (0.00–0.17)** | **<0.01 (0.00–0.01)** |
| *Sympagurus dimorphus* | 0.07 | <0.01 | 0.02 | <0.01 |
| **Brachyura** |  |  |  |  |
| **Goneplacidae** | **6.7 (4.23–9.21)** | **0.79 (0.31–1.58)** | **3.91 (2.12–6.07)** | **0.79 (0.26–1.72)** |
| *Pycnoplax victoriensis* | 2.94 | 0.37 | 0.99 | 0.21 |
| *Neommatocarcinus huttoni* | 1.17 | 0.34 | 0.89 | 0.08 |
| **Majidae** | **0.11 (0.00–0.34)** | **0.09 (0.00–0.32)** | **0.04 (0.00–0.13)** | **<0.01 (0.00–0.01)** |
| *Leptomithrax longipes* | 0.07 | 0.08 | 0.02 | <0.01 |
| **Mysidacea** | **5.51 (3.59–7.74)** | **0.01 (<0.01–0.02)** | **3.47 (2.02–5.28)** | **0.48 (0.17–1.02)** |
| *Mysidetes* spp. | 0.07 | <0.01 | 0.02 | <0.01 |
| Mysidae unidentified | 3.26 | 0.01 | 1.65 | 0.28 |
| **Cumacea** | **0.11 (0.00–0.43)** | **<0.01 (0.00–0.01)** | **0.04 (0.00–0.15)** | **<0.01 (0.00–0.01)** |
| Cumacea unidentified | 0.07 | <0.01 | 0.02 | <0.01 |
| **Amphipoda** | **2.7 (1.42–4.15)** | **<0.01 (<0.01–0.01)** | **1.3 (0.62–2.18)** | **0.09 (0.02–0.21)** |
| *Cystisoma* spp. | 0.07 | <0.01 | 0.02 | <0.01 |
| *Themisto gaudichaudii* | 0.07 | <0.01 | 0.02 | <0.01 |
| Amphipoda unidentified | 1.5 | <0.01 | 0.59 | 0.05 |
| **Isopoda** | **3.57 (2.20–5.21)** | **0.22 (0.06–0.50)** | **3.35 (1.05–8.40)** | **0.32 (0.06–1.08)** |
| *Brucerolis* spp. | 0.98 | 0.07 | 1.01 | 0.05 |
| *Aega monophthalma* | 0.07 | <0.01 | 0.02 | <0.01 |
| Isopoda unidentified | 1.17 | 0.12 | 0.59 | 0.04 |
| **Echinodermata** | **–** | **–** | **–** | **–** |
| Echinoidae | 0.13 | <0.01 | 0.04 | <0.01 |
| **Chondrichthyes** | **0.43 (0.00–1.10)** | **0.04 (0.00–0.14)** | **0.16 (0.00–0.41)** | **<0.01 (0.00–0.01)** |
| **Chimaeriformes** | **0.32 (0.00–0.85)** | **0.98 (0.00–2.87)** | **0.12 (0.00–0.32)** | **0.01 (0.00–0.07)** |
| *Harriotta raleighana* | 0.07 | 0.35 | 0.02 | <0.01 |
| *Hydrolagus novaezelandiae* | 0.07 | 0.21 | 0.02 | <0.01 |
| *H.* spp. | 0.2 | 0.45 | 0.06 | 0.01 |
| Egg case | 0.26 | 0.03 | 0.08 | <0.01 |
| **Osteichthyes** |  |  |  |  |
| **Anguilliformes** | **2.38 (1.27–3.64)** | **11.49 (2.80–21.60)** | **0.87 (0.44–1.34)** | **0.74 (0.14–1.74)** |
| *Bassanago bulbiceps* | 0.07 | 3.87 | 0.02 | 0.01 |
| *Bassanago* spp. | 0.07 | 0.14 | 0.02 | <0.01 |
| *Diastobranchus capensis* | 0.2 | 3.79 | 0.06 | 0.04 |
| Anguilliformes unidentified | 1.17 | 2.68 | 0.36 | 0.19 |
| **Macrouridae** | **16.97 (13.49–20.66)** | **15.94 (9.39–25.70)** | **7.53 (5.71–9.79)** | **10.00 (5.74–17.07)** |
| *Coelorinchus aspercephalus* | 0.26 | 0.78 | 0.13 | 0.01 |
| *C. bollonsi* | 0.2 | 1.03 | 0.08 | 0.01 |
| *C. fasciatus* | 0.33 | 0.55 | 0.13 | 0.01 |
| *C. innotabilis* | 0.2 | 0.29 | 0.11 | <0.01 |
| *C. matamua* | 0.07 | 1.57 | 0.02 | 0.01 |
| *C. oliverianus* | 5.22 | 4.24 | 1.76 | 1.63 |
| *C.* spp. | 2.09 | 1.38 | 0.64 | 0.22 |
| *Lepidorhynchus denticulatus* | 0.52 | 2.17 | 0.17 | 0.06 |
| Macrouridae unidentified | 4.04 | 2.83 | 1.5 | 0.91 |
| **Merlucciidae** | **0.43 (0.00–1.01)** | **10.76 (0.00–25.60)** | **0.28 (0.00–0.67)** | **0.12 (0.00–0.57)** |
| *Macruronus novaezelandiae* trawl | 0.07 | 0.8 | 0.02 | <0.01 |
| *M. novaezelandiae* | 0.46 | 9.84 | 0.19 | 0.24 |
| **Moridae** | **4.76 (2.95–6.64)** | **0.80 (0.31–1.63)** | **1.78 (1.08–2.61)** | **0.31 (0.11–0.68)** |
| *Antimora rostrata* | 0.07 | 0.02 | 0.02 | <0.01 |
| *Notophycis marginata* | 3.72 | 0.57 | 1.14 | 0.33 |
| *Halargyreus johnsonii* | 0.07 | 0.14 | 0.02 | <0.01 |
| *Pseudophycis bachus* | 0.07 | 0.01 | 0.02 | <0.01 |
| Moridae unidentified | 0.13 | 0.06 | 0.04 | <0.01 |
| **Myctophiformes** | **0.22 (0.00–0.66)** | **0.02 (0.00–0.08)** | **0.08 (0.00–0.24)** | **<0.01 (0.00–0.01)** |
| *Lampanyctodes hectoris* | 0.07 | <0.01 | 0.02 | <0.01 |
| Myctophidae unidentified | 0.07 | 0.02 | 0.02 | <0.01 |
| **Notacanthiformes** | **0.54 (0.10–1.25)** | **1.61 (0.18–4.44)** | **0.20 (0.03–0.47)** | **0.02 (<0.01–0.15)** |
| *Notacanthus sexspinis* | 0.33 | 1.46 | 0.09 | 0.03 |
| **Ophidiiformes** | **0.32 (0.00–0.86)** | **1.67 (0.00–5.92)** | **0.12 (0.00–0.31)** | **0.01 (0.00–0.12)** |
| *Genypterus blacodes* | 0.2 | 1.51 | 0.06 | 0.02 |
| **Perciformes** | **1.19 (0.22–2.21)** | **0.38 (0.03–0.73)** | **0.71 (0.12–1.42)** | **0.03 (0.01–0.11)** |
| *Caristius* spp. | 0.07 | 0.11 | 0.02 | <0.01 |
| *Hemerocoetes* spp. | 0.65 | 0.24 | 0.32 | 0.02 |
| **Pleuronectiformes** | **1.19 (0.34–2.23)** | **0.46 (0.08–1.19)** | **0.43 (0.13–0.83)** | **0.03 (0.01–0.10)** |
| *Arnoglossus scapha* | 0.07 | 0.06 | 0.02 | <0.01 |
| *Azygopus pinnifasciatus* | 0.33 | 0.12 | 0.09 | <0.01 |
| Pleuronectiformes unidentified | 0.33 | 0.25 | 0.09 | 0.11 |
| **Scorpaeniformes** | **1.73 (0.74–2.87)** | **4.04 (1.28–8.11)** | **0.63 (0.27–1.05)** | **0.20 (0.03–0.57)** |
| *Ambophthalmos angustus* | 0.07 | <0.01 | 0.02 | <0.01 |
| *Hoplichthys haswelli* | 0.78 | 2.9 | 0.23 | 0.13 |
| *Helicolenus* spp. | 0.2 | 0.48 | 0.06 | 0.01 |
| *Lepidotrigla brachyoptera* | 0.07 | 0.14 | 0.02 | <0.01 |
| *Neophrynichthys* spp. | 0.2 | 0.33 | 0.06 | <0.01 |
| **Stomiiformes** | **0.43 (0.00–1.04)** | **0.24 (0.00–0.76)** | **0.16 (0.00–0.40)** | **<0.01 (0.00–0.03)** |
| *Maurolicus australis* | 0.07 | <0.01 | 0.02 | <0.01 |
| *Photichthys argenteus* | 0.07 | <0.01 | 0.02 | <0.01 |
| *Vinciguerria* spp. | 0.2 | 0.22 | 0.06 | <0.01 |
| **Syngnathiformes** | **0.43 (0.00–0.95)** | **0.51 (0.00–1.78)** | **0.16 (0.00–0.36)** | **0.01 (0.00–0.05)** |
| *Centriscops humerosus* | 0.2 | 0.42 | 0.06 | <0.01 |
| *C.* spp. | 0.26 | 0.04 | 0.09 | <0.01 |
| **Zeiformes** | **0.22 (0.00–0.67)** | **1.28 (0.00–4.36)** | **0.08 (0.00–0.26)** | **0.01 (0.00–0.08)** |
| *Allocyttus niger* | 0.13 | 1.16 | 0.04 | 0.01 |
| *Capromimus abbreviatus* trawl | 0.07 | 0.07 | 0.02 | <0.01 |
| **Discarded fish** | **4.76 (2.79–6.99)** | **29.57 (13.70–48.50)** | **2.29 (1.19–3.71)** | **3.80 (1.09–8.64)** |
| *Macruronus novaezelandiae* | 0.85 | 4.71 | 0.28 | 0.22 |
| *Pseudocyttus maculatus* | 0.07 | 1.08 | 0.02 | <0.01 |
| *Seriolella* spp. | 0.07 | 1.71 | 0.02 | <0.01 |
| *Trachurus* spp. | 1.63 | 18.29 | 0.07 | 1.61 |
| *Thyrsites atun* | 0.07 | 0.41 | 0.02 | <0.01 |
| Discarded fish unidentified | 0.13 | <0.01 | 0.04 | <0.01 |
| **Other groups** |  |  |  |  |
| Natant decapoda unidentified | 3.39 | 0.05 | 1.21 | 0.22 |
| Brachyura unidentified | 1.11 | 0.08 | 0.34 | 0.02 |
| Crustacea unidentified | 9.39 | 0.09 | 2.96 | 1.49 |
| Fish scales | 0.65 | 0.02 | 0.19 | 0.01 |
| Teleost fish eggs | 0.07 | 0.05 | 18.96 | 0.06 |
| Chondrichthyes unidentified | 0.2 | 0.33 | 0.06 | <0.01 |
| Teleosts unidentified | 44.03 | 6.82 | 14.13 | 47.9 |
| Shell fragments | 0.33 | 0.01 | 0.11 | <0.01 |
| Sand | 0.26 | <0.01 | 0.08 | <0.01 |
| Rocks | 0.85 | 0.02 | 4.93 | 0.22 |
| Plastic | 0.07 | 0.01 | 0.02 | <0.01 |
| Human waste (lamb chop) | 0.07 | 0.02 | 0.02 | <0.01 |
| Unidentifiable | 14.87 | 0.11 | 5.37 | 4.23 |

Bold text lines show the point estimates, and 95% confidence intervals estimated by bootstrap resampling, of the percentage frequency of occurrence (%F), percentage weight (%W), percentage number (%N), and percentage Index of Relative Importance (%IRI), for prey grouped at the taxonomic levels used in the multivariate analyses (n = 926). Under each prey group, the normal text lines show the point estimates of the dietary statistics when calculated for all prey types (i.e., at full resolution), with the abiotic material and prey types that could not be allocated to one of the prey groups (so excluded from multivariate analyses) listed at the bottom of the table (n = 1540).
