# Supplementary material for: Diet of Two Large Sympatric Teleosts, the Ling (Genypterus blacodes) and Hake (Merluccius australis)
Source: PLoS One. 2010 Oct 27;5(10):e13647. doi: 10.1371/journal.pone.0013647 (PMC2965093; doi:10.1371/journal.pone.0013647)
Supplement: Appendix S2 — Hake stomach contents from the Chatham Rise. (0.19 MB DOC) [file pone.0013647.s002.doc]

**Appendix S2.** Hake stomach contents from the Chatham Rise.

|  | %F | %W | %N | %IRI |
| --- | --- | --- | --- | --- |
| **Salpida** | **0.33 (0.00–1.42)** | **<0.01 (0.00–0.01)** | **0.23 (0.00–0.96)** | **<0.01 (0.00–0.03)** |
| Salpida unidentified | 0.15 | <0.01 | 0.08 | <0.01 |
| **Mollusca** |  |  |  |  |
| **Teuthoidea** | **7.31 (4.00–10.82)** | **5.17 (1.00–13.19)** | **5.26 (2.80–7.96)** | **1.52 (0.34–3.86)** |
| Cranchiidae | 0.15 | 0.01 | 0.08 | <0.01 |
| *Histioteuthis atlantica* | 0.15 | 0.03 | 0.08 | <0.01 |
| *H.* spp. | 0.45 | 0.56 | 0.25 | 0.01 |
| *Nototodarus* spp. | 0.9 | 1.66 | 0.5 | 0.08 |
| *Todarodes filippovae* | 0.3 | 2.52 | 0.17 | 0.03 |
| *Onykia* (*Moroteuthis) ingens* | 0.75 | 0.12 | 0.41 | 0.02 |
| Teuthoidea unidentified | 1.65 | 0.17 | 0.91 | 0.07 |
| **Crustacea** |  |  |  |  |
| **Copepoda** | **1.00 (0.00–2.54)** | **<0.01 (0.00–0.01)** | **0.92 (0.00–2.44)** | **0.02 (0.00–0.12)** |
| Copepoda unidentified | 0.45 | <0.01 | 0.33 | 0.01 |
| **Euphausiacea** | **3.32 (1.19–5.90)** | **<0.01 (<0.01–0.01)** | **2.29 (0.80–4.18)** | **0.15 (0.02–0.53)** |
| *Nematoscelis megalops* | 0.15 | <0.01 | 0.08 | <0.01 |
| **Natant Decapoda** |  |  |  |  |
| **Campylonotidae** | **0.33 (0.00–1.45)** | **<0.01 (0.00–0.01)** | **0.23 (0.00–1.02)** | **<0.01 (0.00–0.03)** |
| *Campylonotus rathbunae* | 0.15 | <0.01 | 0.08 | <0.01 |
| **Pasiphaeidae** | **18.94 (11.23–26.76)** | **0.11 (0.05–0.19)** | **20.14 (11.09–29.53)** | **7.63 (2.21–17.33)** |
| *Pasiphaea* spp. | 9.76 | 0.11 | 8.51 | 3.26 |
| **Sergestidae** | **5.98 (2.85–9.97)** | **0.02 (0.01–0.04)** | **6.18 (2.99–10.12)** | **0.74 (0.17–2.11)** |
| *Sergestes* spp. | 2.7 | 0.02 | 2.31 | 0.24 |
| Euphausiacea unidentified | 1.8 | <0.01 | 0.99 | 0.07 |
| **Brachyura** | **0.33 (0.00–1.12)** | **<0.01 (0.00–0.01)** | **0.23 (0.00–0.77)** | **<0.01 (0.00–0.02)** |
| Brachyura unidentified | 0.15 | <0.01 | 0.08 | <0.01 |
| **Mysidacea** | **1.00 (0.00–2.90)** | **<0.01 (0.00–0.01)** | **0.69 (0.00–2.01)** | **0.01 (0.00–0.12)** |
| *Neognathophausia ingens* | 0.15 | <0.01 | 0.08 | <0.01 |
| Mysidae unidentified | 0.3 | <0.01 | 0.17 | <0.01 |
| **Amphipoda** | **2.33 (0.63–4.51)** | **<0.01 (<0.01–0.01)** | **1.60 (0.43–3.19)** | **0.07 (0.01–0.29)** |
| *Themisto gaudichaudii* | 0.6 | <0.01 | 0.33 | 0.01 |
| Vibiliidae | 0.15 | <0.01 | 0.08 | <0.01 |
| Amphipoda unidentified | 0.3 | <0.01 | 0.17 | <0.01 |
| **Isopoda** | **0.33 (0.00–1.09)** | **<0.01 (0.00–0.01)** | **0.23 (0.00–0.76)** | **<0.01 (0.00–0.02)** |
| Isopoda unidentified | 0.15 | <0.01 | 0.08 | <0.01 |
| **Chondrichthyes** | **–** | **–** | **–** | **–** |
| Chondrichthyes unidentified | 0.15 | <0.01 | 0.08 | <0.01 |
| **Osteichthyes** |  |  |  |  |
| **Anguilliformes** | **0.33 (0.00–1.41)** | **<0.01 (0.00–0.01)** | **0.23 (0.00–0.98)** | **<0.01 (0.00–0.03)** |
| Nemichthyidae | 0.15 | <0.01 | 0.08 | <0.01 |
| **Beryciformes** | **1.99 (0.31–4.24)** | **4.95 (0.10–11.14)** | **1.37 (0.21–2.94)** | **0.25 (0.01–1.10)** |
| *Beryx decadactylus* | 0.15 | 0.05 | 0.08 | <0.01 |
| *B. splendens* | 0.15 | 1.42 | 0.08 | 0.01 |
| *B. splendens* trawl | 0.3 | 2.62 | 0.17 | 0.03 |
| *B.* spp. | 0.3 | 0.5 | 0.25 | 0.01 |
| *Diretmus argenteus* | 0.15 | <0.01 | 0.08 | <0.01 |
| **Macrouridae** | **46.84 (38.18–56.04)** | **43.63 (31.95–55.81)** | **39.59 (31.33–48.76)** | **77.56 (62.18–87.42)** |
| *Coelorinchus bollonsi* | 0.45 | 1.85 | 0.25 | 0.04 |
| *C. bollonsi* trawl | 0.3 | 1.26 | 0.17 | 0.02 |
| *C. oliverianus* | 5.11 | 3.65 | 2.89 | 1.3 |
| *C. oliverianus* trawl | 0.3 | 0.47 | 0.17 | 0.01 |
| *C.* spp. | 0.45 | 0.09 | 0.25 | 0.01 |
| *Coryphaenoides serrulatus* trawl | 0.15 | 0.19 | 0.08 | <0.01 |
| *Co. subserrulatus* | 0.3 | 0.25 | 0.17 | <0.01 |
| *Co. subserrulatus* trawl | 0.15 | 0.15 | 0.08 | <0.01 |
| *Lepidorhynchus denticulatus* | 13.36 | 29.38 | 8.75 | 19.77 |
| *L. denticulatus* trawl | 1.5 | 4.89 | 0.91 | 0.34 |
| Macrouridae unidentified | 2.4 | 0.46 | 1.65 | 0.2 |
| **Merlucciidae** | **11.96 (7.23–17.39)** | **36.77 (22.50–50.78)** | **10.07 (5.80–15.25)** | **11.15 (4.16–22.60)** |
| *Macruronus novaezelandiae* | 4.95 | 32.33 | 3.39 | 6.87 |
| *M. novaezelandiae* trawl | 0.45 | 3.52 | 0.25 | 0.07 |
| **Moridae** | **1.99 (0.33–4.38)** | **1.00 (0.04–3.32)** | **1.37 (0.22–3.04)** | **0.09 (0.01–0.51)** |
| *Notophycis marginata* | 0.6 | 0.14 | 0.33 | 0.01 |
| *Halargyreus johnsonii* trawl | 0.3 | 0.84 | 0.17 | 0.01 |
| **Myctophiformes** | **3.65 (1.08–6.83)** | **0.30 (0.05–0.69)** | **3.20 (0.96–5.84)** | **0.25 (0.02–0.91)** |
| *Diaphus* spp. | 0.15 | 0.03 | 0.08 | <0.01 |
| *Gymnoscopelus* spp. | 0.3 | 0.08 | 0.25 | <0.01 |
| *Lampanyctodes hectoris* | 0.3 | <0.01 | 0.17 | <0.01 |
| *Lampanyctus* spp. | 0.15 | 0.08 | 0.08 | <0.01 |
| Myctophidae unidentified | 0.75 | 0.04 | 0.5 | 0.02 |
| **Osmeriformes** | **0.33 (0.00–1.46)** | **0.03 (0.00–0.15)** | **0.23 (0.00–0.96)** | **<0.01 (0.00–0.03)** |
| *Nansenia* spp. | 0.15 | 0.03 | 0.08 | <0.01 |
| **Perciformes** | **2.33 (0.63–4.61)** | **4.24 (0.52–9.36)** | **1.60 (0.44–3.17)** | **0.27 (0.01–1.12)** |
| *Epigonus lenimen* trawl | 0.15 | 0.34 | 0.08 | <0.01 |
| *E. telescopus* | 0.3 | 0.17 | 0.17 | <0.01 |
| *E. telescopus* trawl | 0.15 | 0.4 | 0.08 | <0.01 |
| *Cubiceps* spp. | 0.3 | 1.86 | 0.17 | 0.02 |
| *Tetragonurus cuvieri* | 0.15 | 1.38 | 0.08 | 0.01 |
| **Salmoniformes** | **2.33 (0.64–4.39)** | **0.24 (0.02–0.57)** | **1.60 (0.44–3.04)** | **0.09 (0.01–0.31)** |
| *Argentina elongata* | 0.9 | 0.19 | 0.5 | 0.02 |
| *Bathylagus* spp. | 0.15 | 0.04 | 0.08 | <0.01 |
| **Scorpaeniformes** | **1.66 (0.30–3.69)** | **2.80 (0.05–8.33)** | **1.14 (0.21–2.53)** | **0.13 (0.01–0.72)** |
| *Helicolenus* spp. | 0.45 | 0.68 | 0.25 | 0.02 |
| *H.* spp. trawl | 0.3 | 2.06 | 0.17 | 0.03 |
| **Stomiiformes** | **1.66 (0.28–3.50)** | **0.34 (0.03–0.89)** | **1.14 (0.20–2.42)** | **0.05 (0.01–0.22)** |
| *Vinciguerria* spp. | 0.15 | 0.05 | 0.08 | <0.01 |
| *Photichthys argenteus* | 0.3 | 0.17 | 0.17 | <0.01 |
| **Zeiformes** | **0.33 (0.00–1.09)** | **0.37 (0.00–1.25)** | **0.23 (0.00–0.76)** | **<0.01 (0.00–0.05)** |
| Oreosomatidae | 0.15 | 0.36 | 0.08 | <0.01 |
| **Discarded fish** | **0.33 (0.00–1.61)** | **0.03 (0.00–0.14)** | **0.23 (0.00–1.12)** | **<0.01 (0.00–0.04)** |
| *Macruronus novaezelandiae* discard | 0.15 | 0.03 | 0.08 | <0.01 |
| **Other groups** |  |  |  |  |
| Natant decapoda unidentified | 3.3 | 0.04 | 1.98 | 0.26 |
| Crustacea unidentified | 4.35 | <0.01 | 2.89 | 0.49 |
| Fish scales | 38.29 | 0.02 | 34.19 | 50.82 |
| Fishes unidentified | 21.17 | 2.64 | 14.29 | 13.91 |
| Cephalopoda unidentified | 0.15 | <0.01 | 0.08 | <0.01 |
| Shell fragments | 0.75 | <0.01 | 0.41 | 0.01 |
| Sand | 0.15 | <0.01 | 0.08 | <0.01 |
| Rocks | 0.3 | <0.01 | 1.73 | 0.02 |
| Unidentifiable | 9.31 | 0.04 | 5.12 | 1.86 |

Bold text lines show the point estimates, and 95% confidence intervals estimated by bootstrap resampling, of the percentage frequency of occurrence (%F), percentage weight (%W), percentage number (%N), and percentage Index of Relative Importance (%IRI), for prey grouped at the taxonomic levels used in the multivariate analyses (n = 301). Under each prey group, the normal text lines show the point estimates of the dietary statistics when calculated for all prey types (i.e., at full resolution), with the abiotic material and prey types that could not be allocated to one of the prey groups (so excluded from multivariate analyses) listed at the bottom of the table (n = 677).
